# Implementation of Compressed Sensing in Telecardiology Sensor Networks

**DOI:** 10.1155/2010/127639

**Published:** 2010-09-21

**Authors:** Eduardo Correia Pinheiro, Octavian Adrian Postolache, Pedro Silva Girão

**Affiliations:** ^1^Instituto de Telecomunicações, Instituto Superior Técnico, Torre Norte, piso 10, Avenue Rovisco Pais, 1, 1049-001 Lisboa, Portugal; ^2^Instituto Superior Técnico, Universidade Técnica de Lisboa, 1049-001 Lisboa, Portugal; ^3^Escola Superior de Tecnologia de Setúbal, Instituto Politécnico de Setúbal, 2910-761 Setúbal, Portugal

## Abstract

Mobile solutions for patient cardiac monitoring are viewed with growing interest, and improvements on current implementations are frequently reported, with wireless, and in particular, wearable devices promising to achieve ubiquity. However, due to unavoidable power consumption limitations, the amount of data acquired, processed, and transmitted needs to be diminished, which is counterproductive, regarding the quality of the information produced. Compressed sensing implementation in wireless sensor networks (WSNs) promises to bring gains not only in power savings to the devices, but also with minor impact in signal quality. Several cardiac signals have a sparse representation in some wavelet transformations. The compressed sensing paradigm states that signals can be recovered from a few projections into another basis, incoherent with the first. This paper evaluates the compressed sensing paradigm impact in a cardiac monitoring WSN, discussing the implications in data reliability, energy management, and the improvements accomplished by in-network processing.

## 1. Introduction

Given the limitations imposed to cardiac patients, remote cardiac monitoring solutions, specifically wireless and wearable technologies, are of much interest and need to monitor the patient without diminishing his quality of life and are subject to constant improvements [1]. To implement a Telecardiology Sensor Network, a Personal Area Network for cardiac condition monitoring, novel yet dependable architectures may be explored using the technologies of Wireless Sensor Networks (WSNs) [2] by adding a mote to each sensor and replacing any wired connections by the motes' IEEE 802.15.4 radio capabilities. The use of motes is particularly interesting as their small size and meager power consumption is most welcomed by elder or disabled persons, the most demanding users of this type of e-health systems [1]. The most frequently monitored signals, the electrocardiogram (ECG) and photoplethysmogram (PPG), are sampled at moderate frequencies of hundreds of hertz, both for commercial and in research systems [3–9]. However, this order of magnitude is too high for the capabilities of current devices, for instance the Telos mote, so a new concept may be introduced: compressed sensing. 

From the set of physiological signals generally monitored, three are here analyzed, ballistocardiogram (BCG), electrocardiogram and photoplethysmogram, all of which have been scrutinized concerning feature extraction and raw data compression, hence without aiming at compressed sensing implementation [10–12]. These signals provide important knowledge on the cardiovascular system status, and they are acquired and processed by a number of devices [1, 3–19]. For these reasons it is necessary to evaluate quantitatively the outcome of the compressed sensing application to a telecardiology system, namely, the quality of the data generated after the reconstruction of the compressed sensed signal and the benefit in the power consumption of the network. 

Compressed sensing, also known as compressive sensing, has bloom in the beginning of this century, when groundbreaking results and proofs were obtained [20, 21]. The essence of this concept is that, as the signals commonly acquired and processed are not pure noise; there is some transformation in which a fairly sparse representation of the signals is obtained due to redundancies and to the presence of some sort of structure in the data. This paradigm has established that a signal sparse in a transformation may be recovered from a small number of linear measurements in a second transformation incoherent with the first [21, 22]. Consequently, the signal's compressibility is explored, instead of investigating the signal bandwidth, to reduce the number of measurements to acquire and process. These measurements are not samples but signal's representations in the second basis. 

The inverse problem implicitly posed is to recover the signal from the reduced number of measurements, a possible and undetermined system, requiring a criterion to determine the optimal solution of the reconstruction. The classical approach, the use of the *l*_2_ norm, will almost never converge to the intended solution [22], and the use of the *l*_0_ norm is an NP-complete problem [22]. So, the solution with minimum *l*_1_ norm is computed, formulating the so-called basis pursuit problem [23]. The *l*_1_ approach to the problem leads to results similar to the *l*_0_ reconstruction, and it is solvable with complexity polynomial in *N*. Since the user frequently requires a personal computer to interact with the e-health system, the reconstruction algorithms may be implemented at this element, which may also serve as a sink to the sensor network deployed.

In this paper, the implementation of the compressed sensing framework in wireless sensor networks for telecardiology is studied, taking as reference BCG, ECG, and PPG signals. Their compressibility and the appropriateness of compressed sensing implementation employing different basis where these signals are sparse and different interpolation methods has been studied recently [24]. The implementation of such paradigm in WSNs has a meaningful potential, since reduction on the nodes' activity is prospected, diminishing both data acquisition and data transmission loads, which will extend the lifetime of the nodes and of the Telecardiology Sensor Network (TSN) itself. 

Compressed sensing algorithms were applied to simultaneous recordings of BCG, ECG, and PPG obtained from six young and healthy volunteers, with a sampling rate of 1.5 kHz to allow high-resolution analysis. After dividing the data in groups of 2048 samples, downsampled versions of each set were fed into the algorithms to be projected onto an independent and identically distributed (iid) Gaussian set of vectors, thus emulating compressed sensing. The projections were reconstructed using TwIST [25] and compared with the original high-resolution data. Besides estimating the compressibility and the quality of the reconstruction using different wavelet basis, it was also assessed the quality of the reconstructions influence of the loss of packets and the impact on the energy consumption of the devices.

## 2. Defining Concepts

Before detailing the results obtained and the analysis of the compressed sensing paradigm impact in a TSN, the compressed sensing methodology is formally defined and the physiological signals evaluated are described.

### 2.1. Compressed Sensing

The theory of this recently introduced paradigm defines that a time signal, composed by *N* samples, and represented by a vector *x*, is *K*-sparse or *K*-compressible in the basis *ψ*, if *x* can be well approximated by a linear combination of only *K* vectors of *ψ*, where *K* is significantly smaller than the number of samples of *x*. The basis *ψ* is referred to as the sparsity basis, and it is represented by an *N* × *N* matrix in which each column is a basis vector *ψ*_*i*_. If such happens, *N* − *K* expansion coefficients *α*_*j*_, of the representation *x* = *ψ* *α*, are zero or have a negligible value when compared to the small number, *K*, of dominant terms. 

Compressed sensing founding results have proven that a signal *x*, *K*-sparse in *ψ*, may be reorganized from *y*, a vector composed of *M* linear projections of *x* onto another basis Φ, *y* = Φ*x *= Φ*ψ* *α*. The number of projections, *M*, is slightly greater than *K*, however still much smaller than *N*, and Φ has to be incoherent with *ψ*. Incoherency indicates that the elements of Φ cannot represent sparsely the elements of *ψ* and vice versa. Hence, it is necessary to build Φ, the so-called measurement matrix, of dimension *M* × *N*, in such a way that the signal is not damaged by the dimensionality reduction, which is respected if Φ is a random matrix with iid Gaussian entries, or if it is a Rademacher matrix with random ±1 entries.

It is stated in the literature [21, 22] that *x* can be fully described by the *M* measurements taken, that is, the *y* signal. However, since *M* < *N*, the recovery of the original *x* from *y* is ill posed, and the recovery is only possible given the sparsity of *x* in *ψ*. Given that, the recovery of the coefficients *α* is feasible using optimization algorithms under an appropriate norm for the problem definition. Using the *l*_0_ norm to search for the sparsest set of coefficients that generate *y* is NP-complete [22], the use of *l*_2_ norm, despite being the natural approach, will almost never converge to the *K*-sparse solution [22], so the *l*_1_ norm is applied to solve

(1) $$\hat{\alpha }=\arg\;\underset{\alpha }{\min\;}{||\alpha ||}_{l_{1}}\;\text{subject}\;\text{to}\;\Phi \psi \alpha =y.$$

The optimization problem is specified, and the approach taken to solve it was rewriting (1) in (2) and determining the optimum *α* for this problem, equivalent to the basis pursuit, using TwIST [25] (Two-step Iterative Shrinkage/Thresholding), a class of algorithms extending the iterative shrinkage/thresholding algorithms, with *τ* being a regularization parameter. The measurement matrix Φ used was a random Gaussian matrix, and several basis *ψ* were employed, mostly wavelet transforms:

(2) $$\hat{\alpha }=\arg\;\underset{\alpha }{\min\;}\;{||y-\Phi \psi \alpha ||}_{l_{2}}{}^{2}+\tau {||\alpha ||}_{l_{1}}.$$

An example, with signal and transform domain results of an ECG reconstruction from 64 samples using Daubechies 4 is shown in Figure 2, specially highlighting the advances in transform domain characterization of the signal.

### 2.2. Cardiac Signals

**Figure 1:**
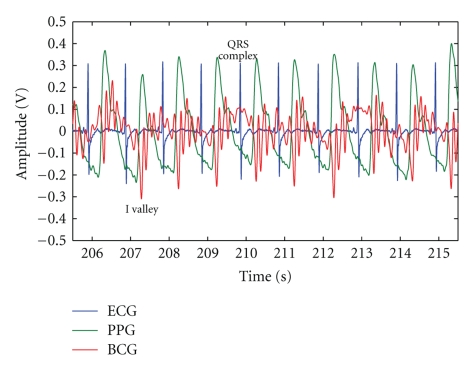
Evolution of the BCG (red), ECG (blue), and PPG (green) signals during 10 seconds, with QRS complex and I valley marked.

Ballistocardiogram is a cardiac signal representing pressure oscillations due to the heart activity obtained recording the body's vibrations by means of a pressure sensor, lately being placed in a chair's back or seat, thus assessing both the BCG and the person's movement [19]. The BCG signal is composed by both systolic and diastolic waves, with the I valley, generated in early systole, being the most noticeable accident of the signal, as seen in Figure 1. The embedding of the sensing apparatus in a chair reduces the patient's involuntary psychophysiological responses related to the measurements' stress, consequently allowing the elimination of important bias sources of cardiologic assessment tests [26]. 

The electrocardiogram signal is a qualitative analysis of the electrical potentials the sinoatrial node generates during the cardiac cycle to stimulate the myocardium and may be acquired using only three chest electrodes if connected to proper amplification and filtering circuitry [19]. The QRS complex is due to ventricular depolarization and is the foremost ECG wave; Figure 1 marks it.

The photoplethysmogram represents the volume changes in an organ due to blood flow. It is a signal similar to the blood pressure waveform and it is commonly implemented also to obtain pulse oximetry, which uses a device that illumines the patient's skin and measures the light transmitted or reflected, with both infrared and visible light acceptable for use [5–7]. 

For the acquisition of the data used in this study, the BCG sensing device was built embedding, in the seat of a normal office chair, a piezoelectric pressure sensor, the ECG was acquired using three chest leads, and the PPG by evaluating index finger absorption of red radiation. The signals obtained are depicted in Figure 1.

An aspect which is necessary to mention is the time sparseness of the BCG and, principally, of the ECG. Both signals are characterized by a main peak, and if no projection is taken on this peak, the transformation will not be representative, as the reconstructed signal will not converge to the typical waveform, limiting the minimum number of measurements to be acquired. To the compressed sensing assessment, the data recorded at 1.5 kHz was divided in groups of 2048 samples, *N*, which constitute the reference waveforms. Downsampled sets, decimated at different powers of 2, from 2^2^ to 2^6^, were fed to the compressed sensing algorithms, resulting in a number of 512 and 32 projections, *M*, which represent an equivalent sampling frequency from 375 Hz down to 23.4 Hz.

## 3. Compression Analysis

Assessment tests of the compressed sensing of these signals were recently published [24]. Symlets 2 and 4, as well as Daubechies 2 and 4, proved to be the most appropriate wavelet transforms for implementing compressed sensing to a downsized number of cardiac signals' measurements. In spite of Biorthogonal 4.4 and 5.5 and their respective reverse, Coiflet transforms provide sparser representations for ECG, PPG, and BCG signals. The reconstruction tests conducted in a set of recordings totalizing 50 minutes showed that the application of Biorthogonal and Coiflet transformations in compressed sensing reconstruction are much worse than the Symlets and Daubechies.

Compressibility assessment, as beforehand stated, was done from a set of 2048 samples of each signal, using (3) to truncate the transformation coefficients to a percentage *p* (1, 5 and 20%) of their maximum, and only then computing the inverse transform

(3) $$\alpha_{i}=\left\{\begin{array}{c} 0,\left|\alpha_{i}\right|\leq p\times \left|\alpha_{\max\;}\right|\\ \alpha_{i},\left|\alpha_{i}\right|>p\times \left|\alpha_{\max\;}\right| \end{array}\right\}.$$

Table 1 presents the number of nonzeroed coefficients and the normalized root mean squared deviation of the truncated inverse transform, th(*x_n_*) calculated using (4). The transformations considered were Haar, Daubechies 2, Daubechies 4, Symlet 2, Symlet 4, Biorthogonal 4.4, Biorthogonal 5.5, Discrete Meyer, Coiflet 4, Reverse Biorthogonal 4.4, Reverse Biorthogonal 5.5 [27], and the Discrete Cosine Transform. Wavelet transforms were computed with

(4) $$\text{nRMS}{\text{D}}_{\text{\%}}=100\sqrt{\frac{1}{n}\overset{2048}{\underset{n=1}{\sum }}{\left[\frac{x_{n}-\text{th}\left(x_{n}\right)}{\max\;\;\left(x_{n}\right)-\min\;\;\left(x_{n}\right)}\right]}^{2}}.$$

Table 1 corroborates the heavy-tailed distribution, for numerous wavelet transforms, of the typical ballistocardiogram, electrocardiogram, and photoplethysmogram signals.

The interpretation of this table has implications in the definition of the inverse problem (2). Since the BCG and the PPG energy and sparsity basis decomposition are different from the ECG characteristic, it is understandable that the optimization problem of the latter should focus more on minimizing the solution size term (larger *τ*), than in the case of the others, where the emphasis should be on the error minimization, given that one knows that the solution will be less sparse. In all the implementations next presented, TwIST's regularization parameter weight was empirically defined according to (5), dependent on the data characteristics

(5) $$\tau =\tau_{\text{coeff}}\times \max\;\;\left|\psi \Phi^{T}y\right|.$$

### 3.1. Lossless Medium Reconstruction

**Figure 2:**
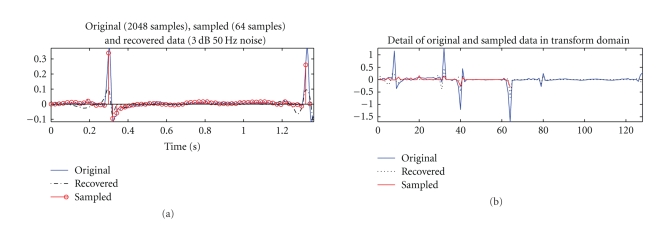
Original (continuous blue), sampled (continuous red), and reconstructed (dashed black) ECG signal from 64 samples. Depiction of time signal (a) and Daubechies 4 wavelet representation with level 4 of decomposition (b).

Table 1 content shows that Coiflet4, Biorthogonal 4.4 and 5.5 and their respective reverses generated sparse representations of the same order of magnitude of other transformations, but the reconstruction tests showed that its application in reconstruction for compressed sensing was much worse than the other wavelet transformations that also generated sparse representations. Daubechies 4 proved to be the one yielding best results. The signal and transform domain results of a reconstruction test are presented in Figure 2, where it is particularly noticeable the improvement in the transform domain characterization. Due to the randomness of the compressed sensing implementation, the signals may have sporadically high nRMSD, even in Daubechies 4. 

**Figure 3:**
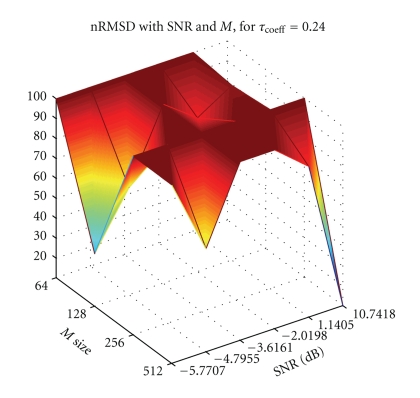
Depiction of SNR and *M* influence in the nRMSD of the ECG waveform reconstruction, for Daubechies 4 wavelet transform and *τ*_coeff_ = 0.24.

Figure 3 exemplifies the dependence exhibited by the nRMSD of the reconstruction of an ECG waveform for different SNRs and number of projections, where 100% error is achieved if the TwIST algorithm does not converge to the original waveform.

### 3.2. Lossy Medium Reconstruction

Compressed sensing implementation in WSN is expected to attain a large benefit. In this section the quantification of this benefit will be estimated, and some issues regarding its application will be addressed. Namely, after the study of an ideal medium without packet losses, the realistic scenario of a medium where packets may be randomly lost is now approached.

In an uncompressed sensing case, when a packet of data containing a measured value is not delivered, the signal will be distorted but the situation is not critical because the lost packet may be estimated from other received packets. When compressed sensing is used, and a data packet with one measurement is lost, it is impossible to estimate its value, due to the random multiplication, and the reconstruction error may irrupt as the algorithm may not converge at all. The worst case occurs when the minimum number of measurements are made, which is 32 in the cardiac signals case. Furthermore, as the ECG is the signal with greater time sparsity, it will be the less affected by random losses, followed by the BCG, with the PPG being the most affected. 

**Figure 4:**
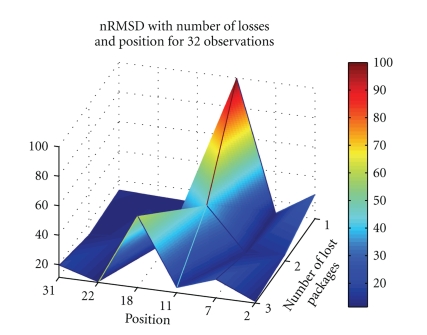
Dependence on number and position of lost packets, for Daubechies 4, *M* = 32, and *τ*_coeff_ = 0.24, of the nRMSD of a reconstructed ECG waveform.

**Figure 5:**
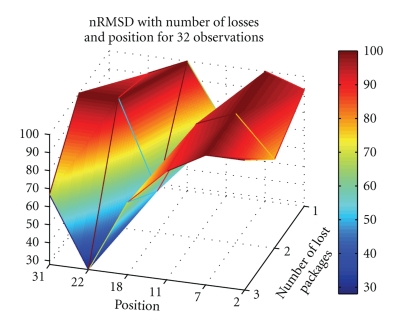
Dependence on number and position of lost packets, for Daubechies 4, *M* = 32, and *τ*_coeff_ = 0.14, of the nRMSD of a reconstructed BCG waveform.

**Figure 6:**
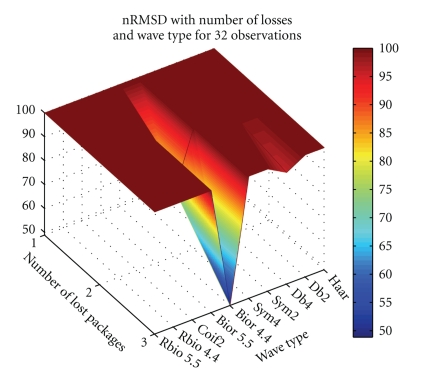
PPG nRMSD dependence on wavelet transform and number of packets lost, for *τ*_coeff_ = 0.14.

Figures 4 and 5 depict nRMSD of the reconstruction when losing one to three packets, in a number of different positions, when using Daubechies 4 wavelet at level 4, for the ECG and the BCG, respectively. Figure 6 depicts the nRMSD increase in the PPG, when losing one to three packets, in random positions in the stream of 32, for different wavelet transformations.

From Figures 4 and 5 it is observable that losing a single packet may induce importance losses, with the nRMSD rising always above 20%–30%. The packet's importance is variable. If it contains information about a major wave detail (as QRS or I valley) the nRMSD can go directly to 100% or to relatively high values, as it can be seen in Figure 4 when the 18th packet is lost. Nevertheless, a packet lost in a moment when the signal possesses low energy will affect less the signal, as it happens when losing packets around the 22nd position in Figure 5.

From Figure 6 it is seen that only one of the transformations attains an nRMSD of 50%, which happens due to the loss of three neighboring packets. All other situations cause TwIST diverging from the PPG waveform.

### 3.3. Cardiac Diseases' Influence

**Figure 7:**
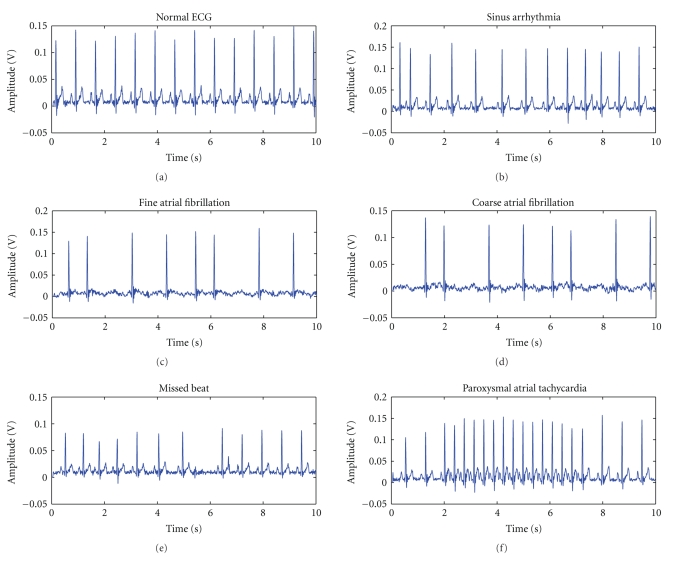
Signals of different cardiac arrhythmias produced by a MPS450 simulator, compared with a normal ECG.

Signals representing five common and well-known supraventricular arrhythmias were gathered, using a Fluke MPS450 patient simulator. The signals were processed using the same methodology as for real patient data. Respiration simulation was programmed, in order to mimic utterly the behavior of a sick patient. The five conditions were fine and coarse atrial fibrillation, paroxysmal atrial tachycardia, sinus arrhythmia, and missed beat. These signals were recorded for different heart rates and amplitudes and are depicted in the following Figure 7.

The signals' compressibility is immediately confirmed from the following Table 2, which presents the results for the top 5% threshold.

Regarding the results on the ECG column of Table 1, it is noticeable an increase in the number of nonzero elements, while the nRMSD is on the same order of magnitude. One significant fact is that the sparsest representations are guaranteed by the Daubechies 2 and Symlet 2 wavelets, while Discrete Meyer and Coiflets are too expensive, without improving the nRMSD. The Biorthogonal wavelets had a higher number of nonzeros, but with an important reduction of the nRMSD. 

In the reconstruction tests it was again verified that Daubechies 4 proved to be generally the transformations yielding best results. Since the signals are arrhythmic ECGs, the results were expected to be analogous, which was confirmed.

## 4. Discussion

### 4.1. Cardiac Data

Table 1 confirms the heavy-tailed distribution, for numerous wavelet transforms, of the typical ballistocardiogram, electrocardiogram, and photoplethysmogram signals. From the compression tests it was verified that Daubechies and Biorthogonal wavelets present sparse descriptions of the BCG, ECG, and PPG signals, and this can be used to reduce the sampling rate down to a minimum of 23.4 Hz, under a reasonable error. The ECG is the signal with most compressibility, followed by the BCG, because of having lower energy than the PPG.

Reconstruction experiments highlighted that TwIST's regularization parameter, *τ*, must be tuned specifically to the signal to be reconstructed, and that, for further optimization of the reconstruction quality, it may be necessary to adjust *τ* even in the same signal, when changing the subject. Regarding overhead in the computations, and in close resemblance with the data of Table 1, ECG is the signal with lower overhead, followed by the BCG, which has close results to the PPG, in the order of few seconds. It was also seen that the number of observations is a governing element on the computation time, while the depth and type of the wavelet transformation is not critical.

**Figure 8:**
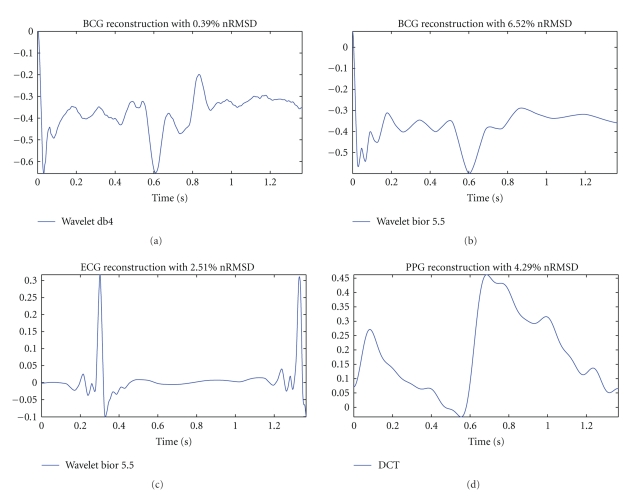
Example of reconstructed signals and the respective nRMSD.

Although the nRMSD is not a very specific indicator of the location of the differences from the original signal, it is a good form of knowing if the major waves are well recomposed. This happens because the cardiac signals approached are known to have one or two major events, thus fail in recomposing the major waves will rise tremendously the nRMSD. Having the principal waves is vital to ensure continuous heart rate monitoring, therefore an nRMSD around 10% is likely to miss details, however it will provide a coarse approximation to the heart rate, and the major waves. If the nRMSD is about 5% the minor waves are likely to have been reconstructed, but the amplitudes and timing synchronisms are not well tuned. When the nRMSD is around 1-2%, the signal is of very good quality. Following Figure 8 illustrates these claims.

It is important to state that, to achieve high resolution in the signals, namely, in the minor waves, and in the ST segment of the ECG, it should be avoided the use of the greater compression ratios. In these cases, the minor waves are poorly defined, or absent, and the establishment of clear relations with the events responsible by T, U, and P waves is not accurate.

### 4.2. Data Delivery

**Figure 9:**
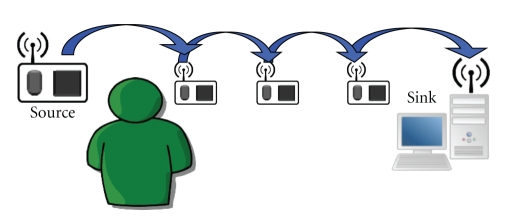
Example TSN, with three intermediate nodes between the source and the sink.

Analyzing the previous results on a lossy scenario, it is verified the tremendous importance, for a WSN dealing with cardiac signals, to transmit all the packets of the compressed data for accomplishing a trustworthy quality of service, especially when the compression is high. This is due to the fact that the compression algorithms can deal with the reduction of the amount of data, but have no ability to hint about what has been lost. In spite of requiring the retransmission of every packet, compressed sensing implementation in a WSN will diminish the amount of data in the network, even for a modest compression rate. For instance, in a TSN with four hops from the sensor to the sink, Figure 9, with a 50% success rate in the transmissions and a protocol that attempts to transmit each packet ten times, assuming packets are saved during this period, each packet will make an average of 7.9531 transmissions to cross the WSN, or 1.9883 transmissions per hop. This result is the outcome of modeling the situation with (6), where #Att is the total number of attempts to transmit a packet, *p* is the success rate, *k* the maximum number of attempts allowed, and *h* the number of hops

(6) $$\#\text{Att}=h\times \overset{10}{\underset{k=1}{\sum }}kp{\left(1-p\right)}^{k-1}.$$

Using compressed sensing in a ratio above 8 : 1, the signal will cross the WSN's four hops with less than one transmission per packet. In shorter networks and/or networks with better connectivity, the gain will be even higher. Furthermore it should be noticed that in an uncompressed sensing scenario, a 50% success rate with four hops will force frequent retransmissions, as the probability of a packet to arrive at the sink will be of 6.25%, thus making impossible to interpolate the time signal from the few packets arrived. Thus, compressed sensing implementation, whenever possible, will be valuable. However, a reliable data transport is mandatory, recurring to negative or positive acknowledgment packets, caching mechanisms or other solution suitable for the application. Discussion on the best protocol for a number of different situations may be found in [28, 29].

**Figure 10:**
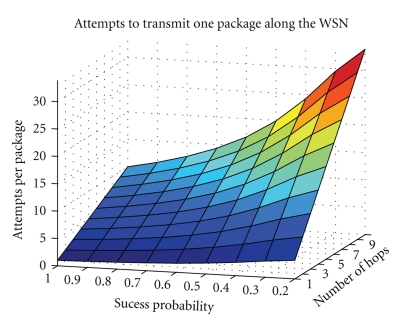
Number of transmissions required for a packet to cross a WSN, with variable length and success probability.

The evolution of the number of attempts to transmit a packet across the Figure 9 TSN, according to (6), for variable number of hops and transmission success rates, is depicted in Figure 10.

### 4.3. Energy Savings

Due to the compressed sensing implementation, the nodes' power consumption is diminished with the reduction of the sampling frequency. Further improvements, optimizing routing, topology, and node sleep periods, or joint optimization techniques [30], may also be explored to increase energy efficiency, restricted to managing the data communications without the introduction of additional delays. 

Related to the reduction on the number of samples to acquire, the overhead and energy dissipated in asleep and awake tasks are reduced as well as the amount of data to send or receive via radio. These two factors are preponderant in the reduction of the power consumption, specially the radio portion, but a third factor, the reduction of the number of samples to process by the node, should also be considered. For different applications, the importance of these factors may shift. For instance, when processing tasks dominate and radio transmissions are negligible, as in [31], the number of packets to represent the waveform is reduced, thus the emitter will send fewer packets, and the receiver will be less time active. Hereby, in such situation, radio activity from both elements is reduced, while also diminishing channel occupancy.

One counter of the compressed sensing application in a TSN is the unbendable requirement of data delivery, which will oblige to an increase of radio usage due to acknowledgement messages and retransmissions. Hence, the compression rate is not proportionally reflected in energy consumption (the Telos mote requires about 20 mA to operate the radio, and 2 mA to use the microcontroller unit). To ensure energy is saved when implementing compressed sensing in a TSN, a minimum compression ratio should be estimated. The boundary for a given number of hops and success rate is obtained from (6), which defines the surface shown in Figure 9.

Energy savings were measured in the device of [5], a sensing node based on a PIC16F877A with a 16 MHz clock, 10 bit ADC, 9 bit USART connected to an RS232-Bluetooth class 1 bridge, and a MAX232 for interface voltage levels translation between the microcontroller and the serial port. Three sensing channels were implemented, acquiring ECG, BCG, and PPG. It was seen that transmitting data increases the power consumption from 0.512 W to 0.745 W. 

Reducing data transmission allows savings of 1/3 of the power expenditure. The power economized per data set, *P*_saved_, resulting from the implementation of compressed sensing in this sensing node is expressed in (7), where *N*/*M* is the compression ratio. The units of *P*_saved_ are Watt per data set

(7) $$E_{\text{saved}}=0.233\frac{N}{M}t.$$

In the concrete case of a sampling frequency of 100 Hz, to send a data set of 1.36 seconds, it would be necessary 136 packets. For an *M* of 32, the power saved would be of 0.990 W.

In spite of naturally allowing energy savings, the implementation of compressed sensing in a TSN must not neglect further optimizations in the network's energy management. The implementation of low power Medium Access Control (MAC) layers based on SMAC [32] for Time Division Multiple Access- (TDMA-) based approaches is important when time features of the signals are also being monitored, as pulse arrival time, an ECG-PPG relation [4–6]. If such happens, ballistocardiography nodes may be put asleep more often, among other possible considerations. To achieve low power operation without TDMA use, two well-known protocols immediately emerge to consideration, BMAC [33], which is more focused in exploiting physical layer, and XMAC [34], which exploits data link layer. Nevertheless, cross-layer MACs such as BoX-MAC [35], may bring benefits to health monitoring WSNs under compressed sensing. Already existent commercial and research solutions [1, 3–19] from the uncompressed sensing world, have idiosyncrasies which may be useful in improving energy management in a TSN.

### 4.4. In-Network Processing

In-network processing of the data gathered by the sensors may be an important way of improving the reconstruction results, particularly in what concerns the reconstruction of the signals' main features. From aggregation functions based on maximum value, the nodes can determine when the major waves of the signals' are present. If the WSN is capable of organizing itself, so that the sampling process concentrates on the moments where the signal is expected to have larger values, then the main waves will have enhanced resolution. The time stamp of the sampling moment must be saved to allow the posterior processing algorithms to deal with this irregularly sampled data. 

In addition, recalling that the period of the cardiac signals varies between 1 and 0.4 seconds, such optimization steps require strict time synchronization in order to achieve the prospected improvements in the signal reconstruction. Due to these specificities, sensor-to-sensor Reference Broadcast Synchronization methods, as described in [36], are particularly well suited.

### 4.5. Network Architecture

Some aspects on how the number of hops from the sensor node to the sink affects the number of transmission attempts were aforementioned, and a depiction was presented in Figure 9, considering that the nodes have caching capabilities, until a maximum number of attempts are attained. It was also detected the importance of delivering every packet. Thus, despite the number of norms available to calculate the network lifetime [37], a TSN implementing compressed sensing collapses when a sensor fails, or when it is partitioned. These constraints are exceptionally demanding, as the sensors' data always has to be delivered. The existence of redundant nodes is profitable and necessary. In order to expand the time to fail, the intermediary nodes should have intelligent and adaptive routing algorithms, energetically efficient and with reasonable overhead. Notwithstanding the independent choice of the best protocol, joint optimization of these factors is a possibility [30]. 

To do personal monitoring two distinct network architectures emerge as the most likely: sensors embedded in the environment surrounding a specific house division, or opportunistic communications using the subject as a data mule, transporting data between isolated parts of the network [38]. Recent developments implemented the three sensing devices in a regular office chair [24]. If this approach is followed, the room must be populated with wireless sensors and the sink (a personal computer necessarily, to hastily reconstruct the signals and to provide a graphical user interface), and add nodes to other rooms, to enable a double or multisink setting, without transporting the chair. However, a more interesting approach for deeper and continuous cardiologic monitoring would be the embedding of ballistocardiographic sensors in the environment in objects such as chairs and wheelchairs, carpets, and beds. This deployment would allow ubiquitous monitoring of daily life activities. It would also help the telecardiology system to contextualize the measurements taken and to accompany the user's condition evolution in a broader time period. The data obtained from the monitored subject is important to carry context-aware information, so the implementation of these devices together allows pervasive monitoring with activity awareness, thus providing better care and reducing false alarms.

Implementing a WSN with numerous nodes and multiple sinks is an important field of research, as the number of acknowledgement messages increase will be unbearable [39]. The modification of the transport protocol, for instance adapting the solutions of [39–41] for reliable first packet dissemination from sink to sensor, would be profound, so probably the most practical solution would be keeping the single sink structure and establish the active WSN sink as a server, so that other devices could externally access for information on the WSN, namely, mobile phones, which are common nowadays [7–9]. When dislocating the physical apparatus to other place, the nearest sink should define itself as a server and provide access for other authorized users, following strictly or with application-required modifications the mule concept [38].

### 4.6. Security

The sensor data transmission is secure and does not require encryption if the values acquired are multiplied by a random floating-point matrix, thus converting the data into white noise. If a Rademacher matrix is used, encryption is necessary again, as the product by such a matrix will only change the signal of the data to transmit, so it is not enough to prevent interpretation of the data in case of intrusion. The reconstruction software has explicit knowledge of the matrix used, as well as the sensor nodes, which have it on memory, so no external access must be allowed when the measurement matrix is being defined and also to the memory sectors where it is saved.

## 5. Conclusions

The implementation of the compressed sensing paradigm in a wireless sensor network for cardiovascular function monitoring was assessed and found to be very profitable. The three cardiac signals studied, BCG, ECG, and PPG, were compressed and recovered with a maximum compression ratio of 64 (thus with a sampling frequency of 23.4 Hz), which is a very high profit situation in a TSN, especially regarding energy savings and network activity reduction. Nevertheless, it was also found that perfect packet delivery is mandatory. Otherwise, the reconstructed signal may be unrepresentative. Failing a packet delivery may invalidate a number of measurements from the network. Thus, in a compressed sensing implementation, to ensure in fact quality of service means to guarantee that all packets are delivered. An expression modeling the compressed-uncompressed boundary was presented, and it was found that, even in such tight delivery constrains, it is easy to achieve the zone where the benefits introduced by compressed sensing overcome the penalties, as compression ratios around 8 will be enough to reduce network traffic and augment network lifetime with high probability, in harsh scenario where only 50% of the transmissions are successful. 

From the results obtained, it was seen that the approach between the two distant worlds of wireless sensor networks and compressed sensing is very interesting and may be further transposed to other areas of patient monitoring. Some difficulties to be expected in future implementations were presented in this paper and some particularities of cardiac signals were addressed. The most important were the tight demands regarding accurate waveform reconstruction and high-resolution in a time-sparse scenario, preoccupations other frequently monitored features, such as respiration or gait, have in lesser degree. Compressed sensing has thus been found as a plausible method to apply in Wireless and Telecardiology Sensor Networks.

## Figures and Tables

**Table 1:** Truncated inverse transform nonzeros and nRMSD%.

*ψ*	BCG						ECG						PPG					
	# NZ			nRMSD			# NZ			nRMSD			# NZ			nRMSD		
	1%	5%	20%	1%	5%	20%	1%	5%	20%	1%	5%	20%	1%	5%	20%	1%	5%	20%
Haar	177	130	127	0.66	1.46	2.08	167	39	10	0.48	1.85	3.74	165	119	87	0.59	1.46	6.30
Db2	136	128	127	0.36	0.78	1.02	121	26	10	0.34	1.68	2.84	129	118	85	0.24	0.89	6.55
Db4	131	128	128	0.28	0.39	0.39	111	22	9	0.31	1.60	2.81	128	118	85	0.18	0.92	6.59
Sym2	136	128	127	0.36	0.78	1.02	121	26	10	0.34	1.68	2.84	129	118	85	0.24	0.89	6.55
Sym4	135	131	131	0.27	0.44	0.44	111	26	12	0.30	1.61	2.77	131	120	85	0.19	1.00	6.54
Bi4.4	134	131	131	0.27	0.36	0.36	109	26	12	0.29	1.57	2.75	131	121	85	0.19	0.96	6.64
Bi5.5	137	133	132	0.28	0.33	0.62	110	29	14	0.33	1.54	2.79	132	122	85	0.20	1.09	7.18
RBi4.4	136	131	131	0.29	0.52	0.52	113	26	12	0.32	1.64	2.90	131	120	85	0.20	1.01	6.49
Rbi5.5	137	133	132	0.30	0.42	0.57	112	26	14	0.31	1.61	2.41	131	122	84	0.20	0.81	6.41
DMey	234	212	204	0.27	0.31	0.40	165	65	47	0.32	1.53	2.43	219	186	96	0.20	1.00	6.53
Coif4	154	135	134	0.28	0.27	1.04	113	28	17	0.28	1.53	2.35	132	125	85	0.20	0.86	6.52
DCT	51	6	1	1.43	9.41	13.31	121	80	48	0.12	0.52	2.12	34	16	3	1.10	4.29	16.95

**Table 2:** 5% Truncated inverse transform nonzeros and nRMSD% for the five arrhythmias.

*ψ*	Sinus		AFib fine		AFib coarse		Parox ATach		Missed Beat	
	# NZ	nRMSD	# NZ	nRMSD	# NZ	nRMSD	# NZ	nRMSD	# NZ	nRMSD
Haar	59	2.03	27	2.10	46	2.27	48	2.17	40	1.60
Db2	43	1.97	27	1.81	42	1.86	54	1.40	27	1.02
Db4	45	1.55	28	1.82	43	1.97	55	1.48	41	0.82
Sym2	43	1.97	27	1.81	42	1.86	54	1.40	27	1.02
Sym4	45	1.63	31	1.88	46	1.98	49	1.46	42	0.63
Bi4.4	46	1.43	33	1.76	42	1.70	54	1.17	36	0.48
Bi5.5	51	1.14	34	1.64	48	1.65	54	0.99	40	0.24
RBi4.4	49	1.71	33	1.72	47	1.87	57	1.14	38	0.20
Rbi5.5	46	1.76	32	1.49	48	1.60	69	0.88	45	0.47
DMey	145	1.93	148	1.86	147	2.19	287	1.68	208	0.76
Coif4	54	1.78	54	2.21	83	2.12	78	1.72	58	0.60
DCT	55	1.37	61	1.09	59	1.12	59	1.78	45	2.44
